# A comparison of COVID-19 incidence rates across six European countries in 2021

**DOI:** 10.2807/1560-7917.ES.2023.28.40.2300088

**Published:** 2023-10-05

**Authors:** Michael Padget, Pauline Adam, Marina Dorfmuller, Clara Blondel, Ines Campos-Matos, Myriam Fayad, Alberto Mateo-Urdiales, David Mesher, Adriana Pistol, Javiera Rebolledo, Flavia Riccardo, Maximilian Riess, Lavinia Cipriana Rusu, Didier Che, Bruno Coignard, COVID-19 Study group, COVID-19 Study Group members, Antonino Bella, Martina Del Manso, Daniele Petrone, Patrizio Pezzotti, Chiara Sacco, Annasara Carnahan, Anine Kongelf, Dieter Van Cauteren

**Affiliations:** 1 Santé Publique France, Saint Maurice, France; 2 COVID Vaccines and Epidemiology, UK Health Security Agency, United Kingdom; 3 Department of Infectious Diseases, Istituto Superiore di Sanità, Rome, Italy; 4 International COVID Team, UK Health Security Agency, United Kingdom; 5 University of Medicine “Carol Davila” Bucharest, Romania; 6 Department of epidemiology and infectious diseases, Sciensano, Brussels, Belgium; 7 Public Health Agency of Sweden, Stockholm, Sweden; 8 National Institute of Public Health Bucharest, Romania; 9 The members of the COVID-19 Study group are listed under Investigators

**Keywords:** COVID-19, incidence, international comparisons, surveillance, Europe

## Abstract

International comparisons of COVID-19 incidence rates have helped gain insights into the characteristics of the disease, benchmark disease impact, shape public health measures and inform potential travel restrictions and border control measures. However, these comparisons may be biased by differences in COVID-19 surveillance systems and approaches to reporting in each country. To better understand these differences and their impact on incidence comparisons, we collected data on surveillance systems from six European countries: Belgium, England, France, Italy, Romania and Sweden. Data collected included: target testing populations, access to testing, case definitions, data entry and management and statistical approaches to incidence calculation. Average testing, incidence and contextual data were also collected. Data represented the surveillance systems as they were in mid-May 2021. Overall, important differences between surveillance systems were detected. Results showed wide variations in testing rates, access to free testing and the types of tests recorded in national databases, which may substantially limit incidence comparability. By systematically including testing information when comparing incidence rates, these comparisons may be greatly improved. New indicators incorporating testing or existing indicators such as death or hospitalisation will be important to improving international comparisons.

## Introduction

Since the beginning of the COVID-19 pandemic, international comparisons of key epidemiological data such as disease incidence rates, case-fatality rates, proportion of severe disease and hospital and intensive care unit (ICU) occupancy rates have been essential in advancing knowledge of the disease, guiding public health responses and assessing the effectiveness of measures [1-4]. These data have also been important elements in the media coverage of the pandemic and in shaping public opinion [5,6]. As countries enter the post-crisis phase of the pandemic and conduct after-action reviews, retrospective comparisons will continue to be important to understand lessons from the pandemic and project future health system needs.

The usefulness of these international comparisons requires a sufficient level of comparability, without which conclusions may be biased or incorrect and lead, in some cases, to less effective public health measures.

In 2020, many European countries looked to their neighbours to learn lessons about COVID-19 responses and benchmark success [7]. Germany identified as an early example to follow based on relatively lower case-fatality rates [8,9], however, the data used came from new information systems with important biases [10]. Early testing capacity was higher in Germany than in many neighbouring countries [11], which increased the proportion of cases detected, including less severe cases, resulting in lower observed case-fatality rates.

International response to the emergence of the severe acute respiratory syndrome coronavirus 2 (SARS-CoV-2) Delta variant (Phylogenetic Assignment of Named Global Outbreak (Pango) lineage designation (B.1.617.2) in 2021 provides another example of the impact of biased data on decisions about public health measures. While the number of cases and deaths in India increased dramatically in the period from mid-March to the end of April 2021 with the spread of this new variant, low testing levels and backlogs of testing results meant that the recorded cases and incidence estimates remained artificially low and below thresholds established by other countries to signal an increased risk [12]. As a result, the wave of reinforced measures in countries worldwide to reduce introduction and transmission of the Delta variant did not start until the end of April. While the Delta variant would likely have spread to all countries eventually, this delay in stringent measures may have helped to accelerate the process.

Many elements can potentially bias the international comparability of epidemiological data including national objectives and surveillance system structures which can impact testing strategy, testing capacity and access, target populations or case definition [13]. To our knowledge, no systematic analysis of these differences or their impact exists.

Disease incidence is one of the most commonly used epidemiological indicators. In order to evaluate the international comparability of COVID-19 case counts and incidence rates in Europe, we undertook a multi-country survey. The study sought to collect data on COVID-19 surveillance system characteristics, identify key differences and suggest methods for improving comparability.

## Methods

### Analysis period and data collection

National COVID-19 surveillance systems are continually evolving and highly dynamic. For the purpose of this study, we limited the analysis to the surveillance systems as they existed in mid-May 2021. Study participants were asked to provide data reflecting their COVID-19 testing strategy and surveillance system at this time point.

Data were gathered using multiple methods. Data on target testing populations, access to testing, types of tests used, testing strategy, data entry and management, statistical approaches to the calculation of case counts and incidence and case definitions were collected using a standard questionnaire followed by semi-structured interviews with participating country representatives. The questionnaire is available in the Supplementary material. Questionnaires and interviews were completed between May and September 2021. Data on the number of COVID-19 tests and positive cases registered between week 13 (29 March to 4 April) and week 30 (26 July to 1 August) 2021 were collected from national public health organisation websites or the European Centre for Disease Prevention and Control (ECDC) and verified by country representatives. This period was chosen to represent the situation around mid-May 2021. These data, along with population figures from Eurostat [14], were used to calculate the average number of tests per 1,000 population per day, the test positivity rate (number of cases divided by the number of tests) and the average 7-day incidence per 100,000 population by week over the study period, and the proportion of RT-PCR among all tests.

Contextual data on the phase of the epidemic and the policies and measures in place were gathered from open sources including government websites and media sources. Media sources were cross-checked against official government sources where possible.

### Country inclusion

Participation in the study was limited to countries in Europe in order to increase the relevance of country comparisons. Epidemiologists at Santé Publique France contacted participants in the World Health Organization (WHO) and ECDC COVID-19 working groups from other European national public health organisations. These participants then identified appropriate epidemiological contacts within their organisation for study participation. Participants from a total of 10 countries were contacted for participation. We sought to include both those countries with significant travel to and from France - typically neighbouring countries - as well as those capable of providing a broader representation of European countries.

Due to high demands on public health agencies during the study period, not all national teams were available for participation. Of the 10 countries invited to participate in the study, six accepted: Belgium, England, France, Italy, Romania and Sweden. Austria, Germany, Greece and Spain did not participate.

### Testing definitions

COVID-19 testing types and logistics can vary widely across countries. This study divided testing into three categories: (I) RT-PCR; (ii) antigenic; (iii) at-home antigenic testing. RT-PCR tests used RT-PCR technology to detect infection. Antigenic tests were defined as those capable of detecting COVID-19 antigens which were conducted with medical supervision, often in a pharmacy or laboratory. At-home antigenic testing used the same detection methods as antigenic tests but testing was conducted by an individual without medical supervision, usually at home. Lateral flow devices, which test for COVID-19 antigens, were used extensively in England in various settings. Depending on the setting, these tests may fit into both the antigenic and at-home antigenic testing categories as defined above. For the purpose of our analysis, we categorised these tests in the antigenic category but have mentioned them in the at-home antigenic testing data for England where appropriate.

## Results

Important differences could be seen across participating countries among factors such as: epidemiological context, public health measures in place, testing rates and types, testing strategy, incidence calculation, data management and case definitions (Figure, Table 1).

**Figure:**
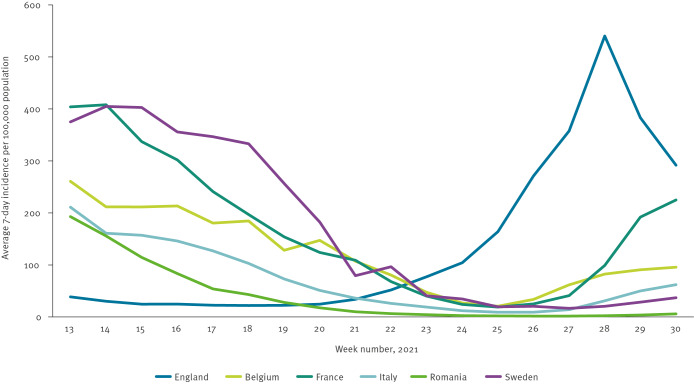
7-day COVID-19 incidence per 100,000 population in six European countries, weeks 13 to 30 2021

Source: National public health websites and European Centre for Disease Prevention and Control (ECDC) reports.

**Table 1:** COVID-19 testing, incidence and epidemic phase in six European countries, weeks 13 to 30 2021

	Average tests per 1,000 population per day^a^	Incidence per 100,000 population per week^a^	Test positivity rate (%)^a^	Proportion (%) of RT-PCR among all tests	Context April–July 2021^b^
**Belgium**	4.3	117	3.9	95	Declining incidence at the end of the third wave before a slow rise in July. Non-pharmaceutical interventions reduced during this period.
**England**	17.4	139	1.1	22	Low incidence before fourth wave in June and July. Non-pharmaceutical interventions reduced during this period.
**France**	4.9	148	4.3	80	Declining incidence at the end of the third wave until quickly rising cases in July. Non-pharmaceutical interventions reduced during this period.
**Italy**	3.8	75	2.8	45	Declining incidence at the end of the third wave before a slow rise in July. Non-pharmaceutical interventions reduced during this period.
**Romania**	1.3	40	4.3	50	Declining incidence at the end of the third wave with stable low rates in July. Non-pharmaceutical interventions remained stable during this period.
**Sweden**	3.3	125	5.4	90	Declining incidence at the end of the third wave before a slow rise in July. Non-pharmaceutical interventions reduced during this period.

^a^ Average across the period from week 13 to week 30 2021.

^b^ Information on non-pharmaceutical interventions represents the general situation over the study period. More detailed information is provided in the Response Measures Database published by European Centre for Disease Prevention and Control (ECDC) [19].

Source: National data, ECDC, participating country representatives, open online sources.

### Context

In Belgium, France, Italy, Romania and Sweden, reported incidence declined between early April and the end of June 2021 following a third wave of infections in each country. Reported incidence in July remained stable in Romania, rose slowly in Belgium, Italy and Sweden and rose quickly in France. In England, reported incidence was stable at a low level from the beginning of April until the beginning of June when a fourth wave began with a peak in mid-July.

During the period from April to July 2021, Belgium, England, France and Italy were progressively lifting non-pharmaceutical intervention measures with most of them removed by mid-May. In Sweden, several measures were in place until the end of May when they began easing. In Romania, few non-pharmaceutical intervention measures were in place during the study period.

### Testing rates and type

Daily testing rates varied widely among countries ranging from 17.4 tests per 1,000 population in England to 1.3 in Romania, with the remaining countries reporting an average of 4.1 per 1,000 population.

Test positivity rate also varied widely between countries. The highest rate was recorded for Sweden with 5.4%, and the lowest was reported by England at 1.1%.

The proportion of RT-PCR testing among all tests varied from 95% in Belgium to 22% in England.

### Testing access and strategy

Overall, access to testing could be grouped into two main categories: (i) some restrictions; or (ii) few restrictions (Table 2). While testing was generally free and without restrictions in France and England during the study period, free testing in other countries was limited to individuals with a prescription or other document or those included in targeted testing groups (Table 2). In France, both RT-PCR and antigenic tests were free during the study period for anyone who wanted them regardless of their status. In England, antigenic tests were free for everyone, while free RT-PCR tests were reserved for those with symptoms or in another target group. In Sweden, free testing was widely available for symptomatic persons and for those in other target groups. In Italy, a doctor’s prescription was necessary for a free test, while in Belgium a QR code (test prescription) provided by a doctor, contact tracer or in some cases by a testing centre was necessary. In Romania, RT-PCR and antigenic tests were free for those with symptoms or in other target groups.

**Table 2:** COVID-19 testing access and strategy, six European countries, 2021

Testing access and strategy								
Country	Overall Testing Access category	Where testing done	Target populations for testing	Testing logistics and payment	Paid testing	Home testing	Non-symptomatic screening outside of target groups	Other
**Belgium**	Some restrictions	RT-PCR: laboratories, and testing centres Antigenic: laboratories, GP’s office.	Those with symptoms, high risk contacts, returning travellers, those targeted in cluster investigation.	A QR code, reserved for target populations, is necessary for a free test. This can be provided by a GP, contact tracer, or in some cases by the testing centre directly.	Anyone without a QR code may pay between 47 and 80 euros for an RT-PCR test.	Individuals may buy at-home antigenic tests in pharmacies.	Some school testing and testing of healthcare workers is organised particularly in the case of cluster investigations. No specific strategy is in place to routinely test non-symptomatic individuals that are not included in targeted testing groups.	The testing system is set up around RT-PCR tests. No need for RT-PCR confirmation of positive antigenic tests performed in laboratories or GP offices. Individuals with positive at-home antigenic tests should perform a confirmation RT-PCR test.
**England**	Few restrictions	RT-PCR: any healthcare setting, at home, in the community, in managed quarantine facilities Antigenic/LFD: anywhere including at home, schools, business or in monitored settings	RT-PCR: Those with symptoms, contacts, travellers from certain countries, ad hoc testing, confirmation of positive LFD if necessary LFD: anyone	RT-PCR tests are free for anyone in target groups and can be performed at listed sites or conducted at home by ordering testing kits to be delivered and returned by mail. LFD tests are always free and can be performed in listed sites, obtained in pharmacies, or ordered online and delivered by mail.	RT-PCR tests for travel related purposes may be purchased at market rates (around 70 euros).	LFD may be conducted at home. RT-PCR samples may be taken at home.	LFD are designed for regular testing of non-symptomatic individuals. National recommendations include using an LFD when engaging in activities that may increase exposure to COVID-19 (social events, etc.). Schools, business, and other structures also have regular non-symptomatic testing using LFD.	Systematic confirmation RT-PCR test is necessary after a positive LFD.
**France**	Few restrictions	RT-PCR: laboratories, hospitals Antigenic: pharmacies, GP’s offices	Those with symptoms, contacts, travellers, ad hoc testing campaigns (e.g. variant investigation) or high risk communal areas (airports, retirement homes). No difference between RT-PCR and antigenic tests.	Anyone can receive a free antigenic or RT-PCR test at testing sites. Appointments at laboratories are often necessary for RT-PCR tests.	All testing is free of charge.	Individuals may buy at-home antigenic tests in pharmacies.	Some screening campaigns in high-risk communal areas (airports, retirement homes, schools) exist as well as among healthcare workers. Individuals may test themselves at any time for any reason for free.	A confirmation RT-PCR test is recommended after a positive antigenic test or a negative antigenic test for symptomatic individuals as well as those with a positive at-home antigenic test.
**Italy**	Some restrictions	RT-PCR: drive-in testing centres, laboratories, hospitals Antigenic: drive-in testing centres, laboratories, hospitals, pharmacies	Those with symptoms, contacts, those testing to end isolation. No official difference between RT-PCR and antigenic tests although differences may exist in practice.	A prescription or another specific authorisation is needed for a free test. These are provided to those in target groups. A prescription can be obtained in person or by phone. Most testing is done in drive-in testing centres.	Anyone can be tested if they pay. Test prices vary by region. RT-PCR may cost around 70 euros. Antigenic tests may cost around 8 to 15 euros	Some home visits by contact tracers are organised to test individuals. Individuals may also buy at-home antigenic tests in pharmacies.	Screening of specific groups exists and depends on regions, (e.g. healthcare workers, schools) In mid-May around 22% of cases nationally were identified through screening activities.	Regional differences between testing type and strategy exist. Individuals with a positive at-home antigenic test should perform a confirmation RT-PCR or antigenic test in an authorised facility.
**Romania**	Some restrictions	RT-PCR: laboratories, hospitals Antigenic: pharmacies, GP’s office	Those with symptoms, those testing to end isolation, symptomatic individuals with a negative antigenic test. Those included in medical risk groups (e.g. oncology, dialysis) if accessing the health system.	Testing is free for those who fall into the target groups.	For those who do not qualify for a free test, RT-PCR may be conducted for 50 euros and antigenic tests for 25 euros	Individuals may buy at-home antigenic tests in pharmacies.	Screening on hospital admission is conducted as well as routine screening of health and social workers and residents in long-term care units.	Individuals with positive at-home antigenic tests should perform a confirmation RT-PCR test.
**Sweden**	Some restrictions	RT-PCR: drive-in testing centres, hospitals, testing centres, home delivery of tests Antigenic: hospitals, primary healthcare facilities including elderly care	RT-PCR: Symptomatic individuals, contacts, travellers, ad hoc studies, those with scheduled surgery, those confirming a positive antigenic test Antigenic: specific situations when a quick result is needed including outbreaks or other special situations	Free tests are available for those within the target groups and available through testing centres or home-delivery of self-sampling kits. Antigenic tests used within the healthcare system are also free for target groups.	Anyone outside of targets groups can pay for RT-PCR or antigenic testing (30–100 euros).	In some regions, most sampling for RT-PCR tests takes place at home through a delivery system. Individuals may also buy at-home antigenic tests in pharmacies.	Outside of contact testing and special studies, no specific efforts are made for non-symptomatic testing in the community. National point prevalence studies using PCR have been done periodically throughout the pandemic to estimate prevalence nationally. Some regional screening is done to identify cases among healthcare workers.	RT-PCR tests are generally not recommended within 6 months of having had a positive RT-PCR result in the community. A positive antigenic test or at-home antigenic test should be confirmed with an RT-PCR test.

GP: general practitioner; LFD: lateral flow device.

In all countries, paid testing was available for those not eligible for free testing. The cost varied among the countries and according to the type of test. Antigenic tests cost 8–15 euros in Italy, 25 euros in Romania and around 30 euros in Sweden. RT-PCR testing cost 47–80 euros in Belgium, 70 euros in Italy and England, 50 euros in Romania and up to 100 euros in Sweden. Both RT-PCR and antigenic tests were free in France.

All countries permitted the purchase of at-home antigenic tests in pharmacies.

Across all countries, the testing locations for antigenic tests were at least partially different from locations for RT-PCR tests. The most common locations for antigenic tests were pharmacies and general practitioner’s (GP) offices, while RT-PCR tests were available in laboratories, hospitals or testing centres. In Belgium and Sweden, medically-assisted antigenic tests were not available in pharmacies. Only in Sweden and Italy were antigenic tests available in hospitals. Target groups for testing were relatively homogeneous across countries with the exception of Romania where persons identified as a contact were not included in target groups. In Belgium, only high-risk contacts were included among target groups.

Systematic screening of non-symptomatic individuals outside target groups was conducted differently between the countries in our study. In Belgium, this type of testing was conducted in schools, in long-term care facilities (LTCF) and within the healthcare workforce, notably in the case of clusters. Similarly, schools, healthcare workers and residents/staff in long-term care facilities were targeted in Italy, although differences in implementation existed across regions (NUTS-2 subnational level). Romania targeted non-symptomatic individuals in hospitals (hospital admissions, health and social personnel) and LTCF. Sweden reported testing non-symptomatic individuals in the case of contact-tracing and as a part of national point prevalence studies. France tested non-symptomatic individuals in high-risk communal areas such as airports as well as in schools and among healthcare workers. Reinforced non-symptomatic testing was facilitated by temporary testing structures. In England, systematic screening was conducted in both schools and businesses. In addition, regular screening was recommended to all individuals, especially prior to participation in any events with a high risk of viral transmission.

In Italy, home visits could be scheduled by contact-tracers in order to conduct an RT-PCR test.  Both Sweden and England made it possible for individuals to order RT-PCR tests, conduct the sampling at home themselves and then send the sample to a laboratory for testing.

### Data entry

Most studied countries reported having a manual testing data entry system for at least some tests results (Table 3).

**Table 3:** COVID-19 testing data entry, exhaustivity of data, data logistics and case definition, six European countries, 2021

Testing data entry and exhaustivity					Data logistics	Case definition
Country	RT-PCR tests	Antigenic tests	At-home antigenic tests	Missing tests		
**Belgium**	Testing data are entered first into local databases by laboratory personnel then sent electronically to one central data warehouse.	Testing data are entered first into local databases by laboratory personnel or GPs then sent electronically to one central data warehouse.	Positive at-home antigenic tests may be reported to the national database if they accompany a positive RT-PCR confirmation test. Positive at-home antigenic tests may also be included in the national database if a physician reports it as a valid antigen test. Otherwise, these tests are not reported.	Results of at-home antigenic tests generally are not recorded in the national database.	RT-PCR and antigenic tests are entered into the same national database. A unique national person ID is used to remove multiple records for the same patient.	Confirmed case is a person, symptomatic or not, with a laboratory result confirming infection with SARS-CoV-2, for example molecular amplification (RT-PCR, RT-LAMP) or antigen test. A positive test is counted as a new case only if no other positive test has been recorded for the same person (ID) within the last 90 days.
**England**	Testing data are entered into national database by laboratory personnel.	Depending on where the test is done, this can be done manually by the individual who self-administered the test, the school, workplace, healthcare facility or testing site where testing took place, via an online platform.	If any individual conducts a lateral flow test at home, they are responsible for recording the result in the national database via an online platform.	Lateral flow test results should be recorded by the person/organisation performing them. An unknown proportion of these tests are not reported.	RT-PCR and antigenic test results are entered into the same national database. A unique national person ID is used to remove multiple records for the same patient.	A positive RT-PCR or LFD test is considered a confirmed case. If a person has more than one positive test, they are only counted as one case regardless of the time between tests. If an RT-PCR test is negative in the 72 hours following a positive LFD, the case is not counted.
**France**	Testing data are entered into local databases by laboratory personnel and sent electronically to a national database.	Manual entry into national database is done by health professionals (nurses, GPs, pharmacists, dentists).	This information is not entered in the national database directly. RT-PCR confirmation tests following positive at-home antigenic test include free text for a specific code which indicates that the RT-PCR is following a positive at-home antigenic test. This information is not analysed at this point. A website is very recently available for results of at-home antigenic tests for entry by individuals but this is not linked to a national database.	Some antigenic tests may be not be recorded by health professionals and no at-home antigenic tests are recorded nationally.	RT-PCR and antigenic test results are entered into the same national database. A pseudonym using name and date is used to remove multiple records for the same patient when counting new cases.	Confirmed case is a person, symptomatic or not, with a laboratory result confirming infection with SARS-CoV-2, for example molecular amplification (RT-PCR, RT-LAMP), antigen test or serology. A positive test is counted as a new case only if no other positive test has been recorded for the same person within the last 2 months.
**Italy**	Testing data are recorded by the person conducting the test (usually pharmacy or laboratory). These results are then centralised at the local, regional and national level. Case data follow the same pathway but start with a physician or primary health provider.	Testing and case data are provided by the regions and aggregated at the national level.	At-home antigenic tests are not recorded in national databases.	Results of at-home antigenic tests are not recorded in national databases.	RT-PCR and antigenic tests are included in an individual surveillance database. A patient number created for this database is used, along with other checks, to remove multiple test results.	A case is any person with a positive RT-PCR or antigen test for SARS-CoV-2. Depending on the region a confirmation RT-PCR test is required for positive antigenic tests. A positive test is not considered a new case if a positive test was recorded in the previous 90 days.
**Romania**	Testing data are recorded by laboratory personnel then uploaded to the national database or entered directly into the national database by the public health authority personnel.	Testing data are recorded by laboratory personnel, medical centres, or GPs then uploaded to the national database or entered directly into the national database by the public health authority personnel.	At-home antigenic tests are not recorded in national databases.	95% of RT-PCR and antigenic tests are registered in national databases. Results of at-home antigenic tests are not recorded in national databases.	RT-PCR and antigenic tests are entered into the same national database. A unique confirmation number created for this database is used to remove multiple records for the same patient.	A confirmed case is anyone who meets the laboratory criteria: detection of SARS-CoV-2 nucleic acid or antigen in a nasopharyngeal sample.
**Sweden**	Testing data are entered into the national database by the diagnostic laboratory.	Testing data are entered into the national database by either the treating GP or the diagnostic laboratory.	At-home antigenic test are not recorded in national databases.	Some privately performed antigenic tests data may not be included in the national database (e.g. workplace screening). At-home antigenic tests are not recorded in the national database. Positive at-home antigenic tests should have a confirmation RT-PCR test.	RT-PCR and antigenic tests are entered into the same national database. A unique national person ID is used to combine multiple records for the same patient.	A confirmed case must meet one of the following criteria: (i) detection of SARS-CoV-2 nucleic acid (e.g. RT-PCR test but theoretically also other tests such as RT-LAMP); (ii) isolation of SARS-CoV-2 virus; (iii) detection of SARS-CoV-2 antigen. A positive test within 12 months of another for the same person is not considered a new case.

GP: general practitioner; LFD: lateral flow device; SARS-CoV-2: severe acute respiratory syndrome coronavirus 2.

Outside of England, no country recorded the results of at-home antigenic testing into national databases. However, RT-PCR test confirmation for a positive result was required or recommended in these countries. The results of lateral flow device tests performed as at-home antigenic testing in England were to be recorded by the individual conducting them on a website. In France and Belgium, some information on at-home antigenic testing results was collected if an individual conducted an RT-PCR confirmation test following a positive at-home antigenic test. The collection of this information, however, was not systematic. In France, a website was also available for individuals to report the results of an at-home antigenic tests, although this was not linked to the national database.

### Data management and missing data

All studied countries had one central database for all testing results. For patient identification and elimination of double counting in the context of RT-PCR confirmation tests, all countries had a single patient identifier.

Overall, countries indicated that unreported data for RT-PCR or antigenic testing was rare. The largest potential source of unreported testing came from at-home antigenic tests that were not recorded in national databases. However, all countries recommended a confirmatory RT-PCR test after a positive at-home antigenic test. No country was able to estimate the total number of at-home antigenic tests that were conducted or potentially missed in national databases or those that were followed by a confirmation RT-PCR. In England, individuals were required to report at-home antigenic test results on an online platform but the proportion of those that may have not done so is unknown.

### Case definition

All countries included persons presenting a positive test result (RT-PCR or antigenic) as a positive COVID-19 case.

However, some differences did exist between countries during the study period regarding the definition of reinfection which can impact how multiple positive tests for an individual are recorded. In both Belgium and Italy, a period of 90 days between positive tests for the same individual was necessary for a new case to be counted. This period was 60 days in Italy if genotypic information was available showing that two infections were caused by different viruses. In France, this period was 60 days and in Sweden 12 months. England did not refer to a time limit and mentioned in its case definition that an individual could only count as a single positive case. Some of these definitions have changed since data were collected.

## Discussion

Our study showed important differences in access to testing, types of tests used and the recording of test results which were associated with substantial differences in testing rates. These differences may bias direct international comparisons of case numbers and incidence rates.

One principal difference across countries impacting reported incidence rates and case counts was average testing rates. Testing rates are the fundamental element in case detection and incidence calculation. Increased testing including systematic screening will generally result in more cases being detected so the widely varying rates observed pose a potentially serious obstacle to comparability. Increased testing may also reduce the positivity rate particularly in the case of increased asymptomatic screening.

Testing rates can be highly variable according to the national policy and disease burden, with cases and testing rates being positively correlated. A higher or lower testing rate is therefore not sufficient to bias incidence comparisons by itself, as long as other indicators such as positivity rates remain relatively comparable and there are not substantial differences in populations being tested. This highlights the importance of considering positivity rates or other indicators relevant to testing rates when conducting incidence comparisons. In our study, the large differences observed between countries in both positivity rate and testing rate suggest that incidence rates were not directly comparable.

Some of the differences in testing rates may be explained by differences in access to free testing. The highest testing rates were observed in England, where antigenic tests were free and easily accessible to all. The second highest testing rates were observed in France where both RT-PCR and antigenic tests were free for everyone. The lowest testing rates were reported in Romania where contacts were not eligible for free testing contrary to other countries in the study.

Systematic screening of asymptomatic individuals outside of target groups also played an important role in testing and positivity rate differences. In Sweden, testing of asymptomatic individuals was primarily used in the case of clusters and contact tracing, which may help explain the relatively low testing rates and high positivity rates observed relative to other countries where asymptomatic screening was more common. Contact tracing efforts were not well described in our study but differences across countries, including the use of dedicated phone applications, may have contributed to testing rate differences. In England, frequent testing of non-symptomatic individuals using lateral flow devices was actively encouraged. The impact of this strategy is reflected in the substantially higher testing rate and lower positivity rate in England.

Screening of asymptomatic individuals also had an important impact on case detection. Although no comparable information was available on the proportion of symptomatic vs non-symptomatic testing in the countries studied, Italy reported that over 20% of cases detected came via routine testing of asymptomatic individuals. A recent review study estimated the proportion of asymptomatic COVID-19 infections at over 40% [15].

Testing rates and positivity rates may also be impacted by which tests are recorded as well as the population’s adherence to guidelines. Outside of the recording of lateral flow devices conducted at home in England, at-home antigenic tests were not recorded in national databases. The lack of data on these tests means the true number of tests is unknown across countries. These missing data may bias the numerator when calculating testing rates and the denominator when calculating positivity rates. A confirmation RT-PCR was recommended or required for individuals with a positive at-home antigenic test in all countries, but no information on adherence to these policies was available. Using RT-PCR tests to confirm positive at-home antigenic testing may increase positivity rates of RT-PCR tests, although during the study period at-home antigenic tests were relatively new and little used in most countries and the impact of these test on RT-PCT positivity rates was likely limited.

In addition to the wide range of testing rates, the types of tests used may also impact comparability as the relative sensitivity and specificity of antigenic and RT-PCR tests differ [16,17]. A country which used the more sensitive RT-PCR test such as Sweden may have a higher positivity rate vs another country with a higher proportion of antigenic testing. In England, the high proportion of antigenic testing may in part explain the relatively low positivity rate, although testing strategy and testing rate likely played a more important role.

Overall, data systems and data management were similar across countries and likely created only small hurdles to comparability. One notable exception was in England where all results for at-home antigenic tests were recorded in national databases.

Case definitions were generally similar, although some variation did exist between countries in the definition of a reinfection. At the time of this study, cases of reinfection were uncommon so these differences were likely to have had little impact on comparability.

While our study identified several important differences in surveillance systems, it also had several limitations. First, our study collected data for a single time point around May 2021. Surveillance systems can be highly dynamic and our study was not able to consider these changes.

Second, our study focused on the systematic features of surveillance systems but did not consider other possibly important factors in system performance such as population adherence to recommendations. Adherence can be differentially impacted by cultural or socioeconomic factors, country or region, symptom severity or other factors which may create important biases in testing results. Other factors outside the surveillance systems that were not measured include current control measures in place such as school closings and the physical availability of tests. However, country representatives did not feel that testing was impacted by a lack of available tests during the study period.

Our study was also limited to six European countries. International comparisons are not limited to European countries and analysing international differences in surveillance systems and indicator comparability is important to draw global lessons. Regional differences within countries were also not analysed, although these differences may impact surveillance system organisation, performance and testing uptake.

Reporting delays and how they are handled when calculating incidence rates were not well detailed in our study despite the fact that these may impact comparability.

Lastly, our study was limited to the consideration of case detection and incidence calculation. Understanding the comparability of other indicators such as hospitalisation or death rates is also important to conduct meaningful country comparisons.

Our study highlighted the difficulty of direct country comparisons of COVID-19 incidence rates and showed important differences in testing rates due in part to systematic differences in surveillance systems. While knowledge of these differences is useful when comparing countries, completing an in-depth surveillance system analysis every time a direct country comparison is undertaken is an unrealistic objective. However, many of the systematic differences such as testing access, target populations and data collection were ultimately reflected in the testing and positivity rates in our study. Systematically including testing information and positivity rates in international incidence comparisons could be a first step towards reducing the biases in these comparisons created by surveillance system differences. This approach may be particularly important for institutions providing publicly available data and infographics (maps, charts etc.) which directly compare incidence rates.

Along with simply presenting testing rates alongside incidence when comparing countries, other possible solutions may include the creation of new indicators, although more research is needed in this area to create robust and useful measures. Analysing the relationship between incidence and excess deaths may also provide important insight as might indicators including hospitalisations, deaths and hospital and ICU occupancy rates. Other relevant indicators from outside of the health system may include sewage surveillance data.

Important biases exist regarding asymptomatic testing as well as the use and recording of at-home antigenic tests. Enhancing incidence comparability in Europe may require better harmonisation of recommendations and practices at the European Union (EU) level on these aspects through the work of ECDC, WHO Regional office Europe or EU countries.

Other approaches to comparisons that do not rely on testing may include syndromic surveillance. Trends in incidence may also be relevant particularly when looking at the impact of control measures or other cases where the precise number of infections is not necessary.

## Conclusions

Our study showed the fundamental difficulty of comparing COVID-19 incidence across countries within Europe. Despite potential biases, these comparisons remain important as we move towards endemic COVID-19 circulation and post-pandemic surveillance. Systematically including the testing information when comparing incidence rates, taking into account other indicators or using representative, internationals surveys such as the COVID-19 Infection Survey in the UK [18] may help to improve these comparisons and the valuable lessons they provide.

## COVID-19 Study Group members

Antonino Bella, Martina Del Manso, Daniele Petrone, Patrizio Pezzotti, Chiara Sacco (Department of Infectious Diseases, Istituto Superiore di Sanità, Rome, Italy), Annasara Carnahan, Anine Kongelf, (Public Health Agency of Sweden, Stockholm, Sweden), Dieter Van Cauteren (Department of epidemiology and infectious diseases, Sciensano, Brussels, Belgium).

## Supplementary Data

33000 Supplementary Material
